# Clinical commissioning and use of the Novalis Tx linear accelerator for SRS and SBRT

**DOI:** 10.1120/jacmp.v13i3.3729

**Published:** 2012-05-10

**Authors:** Jinkoo Kim, Ning Wen, Jian‐Yue Jin, Nicole Walls, Sangroh Kim, Haisen Li, Lei Ren, Yimei Huang, Anthony Doemer, Kathleen Faber, Tina Kunkel, Ahssan Balawi, Kimberly Garbarino, Kenneth Levin, Samir Patel, Munther Ajlouni, Brett Miller, Teamor Nurushev, Calvin Huntzinger, Raymond Schulz, Indrin J. Chetty, Benjamin Movsas, Samuel Ryu

**Affiliations:** ^1^ Department of Radiation Oncology Henry Ford Health System Detroit MI; ^2^ Department of Radiation Oncology Duke University Medical Center Durham NC; ^3^ Varian Surgical Sciences Varian Medical Systems, Inc. Palo Alto CA USA

**Keywords:** Novalis Tx, commissioning, stereotactic radiosurgery, stereotactic body radiation therapy

## Abstract

The purpose of this study was to perform comprehensive measurements and testing of a Novalis Tx linear accelerator, and to develop technical guidelines for commissioning from the time of acceptance testing to the first clinical treatment. The Novalis Tx (NTX) linear accelerator is equipped with, among other features, a high‐definition MLC (HD120 MLC) with 2.5 mm central leaves, a 6D robotic couch, an optical guidance positioning system, as well as X‐ray‐based image guidance tools to provide high accuracy radiation delivery for stereotactic radiosurgery and stereotactic body radiation therapy procedures. We have performed extensive tests for each of the components, and analyzed the clinical data collected in our clinic. We present technical guidelines in this report focusing on methods for: (1) efficient and accurate beam data collection for commissioning treatment planning systems, including small field output measurements conducted using a wide range of detectors; (2) commissioning tests for the HD120 MLC; (3) data collection for the baseline characteristics of the on‐board imager (OBI) and ExacTrac X‐ray (ETX) image guidance systems in conjunction with the 6D robotic couch; and (4) end‐to‐end testing of the entire clinical process. Established from our clinical experience thus far, recommendations are provided for accurate and efficient use of the OBI and ETX localization systems for intra‐ and extracranial treatment sites. Four results are presented. (1) *Basic beam data measurements:* Our measurements confirmed the necessity of using small detectors for small fields. Total scatter factors varied significantly (30% to approximately 62%) for small field measurements among detectors. Unshielded stereotactic field diode (SFD) overestimated dose by ~ 2% for large field sizes. Ion chambers with active diameters of 6 mm suffered from significant volume averaging. The sharpest profile penumbra was observed for the SFD because of its small active diameter (0.6 mm). (2) *MLC commissioning:* Winston Lutz test, light/radiation field congruence, and Picket Fence tests were performed and were within criteria established by the relevant task group reports. The measured mean MLC transmission and dynamic leaf gap of 6 MV SRS beam were 1.17% and 0.36 mm, respectively. (3) *Baseline characteristics of OBI and ETX:* The isocenter localization errors in the left/right, posterior/anterior, and superior/inferior directions were, respectively, −0.2±0.2 mm, −0.8±0.2 mm, and −0.8±0.4 mm for ETX, and 0.5±0.7 mm, 0.6±0.5 mm, and 0.0±0.5 mm for OBI cone‐beam computed tomography. The registration angular discrepancy was 0.1±0.2°, and the maximum robotic couch error was 0.2°. (4) *End‐to‐end tests:* The measured isocenter dose differences from the planned values were 0.8% and 0.4%, measured respectively by an ion chamber and film. The gamma pass rate, measured by EBT2 film, was 95% (3% DD and 1 mm DTA). Through a systematic series of quantitative commissioning experiments and end‐to‐end tests and our initial clinical experience, described in this report, we demonstrate that the NTX is a robust system, with the image guidance and MLC requirements to treat a wide variety of sites — in particular for highly accurate delivery of SRS and SBRT‐based treatments.

PACS numbers: 87.55.Qr, 87.53.Ly, 87.59.‐e

## I. INTRODUCTION

There exists a large body of literature demonstrating the efficacy of stereotactic radiosurgery (SRS)^(^ ^1^ ^–^ ^3^ ^)^ and stereotactic body radiation therapy (SBRT).^(^ ^4^ ^–^ ^8^ ^)^ These advanced modalities deliver a large dose either in a single or small number of treatment fractions, also known as hypofractionated radiation therapy.^(^ ^8^ ^)^ SRS and SBRT treatments require extremely conformal dose distributions to deliver an ablative fractional dose to the target, while limiting dose as low as possible to the nearby normal tissues and the critical organs at risk. It is imperative that the linear accelerator system includes the necessary tools to deliver the highly conformal planned dose distributions as accurately and precisely as possible. One such modern system is the Novalis Tx (Varian Medical Systems, Palo Alto, CA and BrainLAB, Feldkirchen, Germany). Novalis Tx is a marriage of two well‐established linear accelerator platforms from two separate vendors: the classic Novalis (BrainLAB, Feldkirchen, Germany) and the Trilogy (Varian Medical System, Palo Alto, CA). Novalis Tx includes the ExacTrac (available previously on the Novalis unit) and the on‐board imager (present on the Trilogy unit) localization systems. The equipped high‐definition multi‐leaf collimator (HD120 MLC) is specially designed for small SRS targets, having fine leaves centrally, as well as for regular sized targets.

The ExacTrac (ETX) system is an image‐based guidance system consisting of two infrared (IR) cameras, dual X‐ray imagers, and a robotic couch. The IR cameras guide the initial patient setup by monitoring 4–5 external IR markers attached to the patient surface. Two kilovoltage (kV) planar X‐ray images are acquired and coregistered with the 3D CT images, using a fully automatic intensity‐based 3D/2D image registration algorithm, which is also often referred to as “6D fusion”.^(^ ^9^ ^)^ The registration algorithm provides the table corrections in three translations and three rotations. The robotic couch is then driven accordingly to the target position automatically. Accurate localization using the ETX system has been demonstrated in several studies.^(^ ^10^ ^–^ ^13^ ^)^ However, the application is generally limited to bony alignment because soft tissue is often not visible on planar X‐ray images. The on‐board imager (OBI) includes a kV imaging panel that is mounted on the linear accelerator gantry, orthogonally to the megavoltage (MV) therapy beam axis. Therefore, it can acquire planar X‐ray images at any angle. A series of angular projection images can be reconstructed to a volumetric cone‐beam computed tomography (CBCT) image,^(^ ^14^ ^–^ ^18^ ^)^ which has become a popular modality for image‐guided radiation therapy (IGRT).^(^ ^19^ ^)^ The major benefit of this volumetric imaging is soft tissue conspicuity. CBCT images are routinely used for prostate localization,^(^ ^20^ ^)^ as well as for the targets in the lung and abdominal area. Additionally, Novalis Tx also has a MV electronic portal imaging device (EPID), PortalVision. It is mounted at the treatment beam exit, and it is used for routine patient setup verifications, as well as dosimetric verification and linear accelerator quality assurance tasks.^(^ ^21^ ^–^ ^25^ ^)^ The technical specifications of imaging detectors are listed in Table 1.

**Table 1:** Technical specifications of imaging detectors mounted on NTX.

	*MV Imager (EPID)*	*OBI kV imager*	*ExacTrac X‐ray (ETX)*
Model	PortalVision aS1000	Paxscan 4030CB	XRD 840 AN
Type	aSi	aSi	aSi
Pixel Matrix (Pixel Pitch)	1024×768, (0.392 mm)	2048×1536, (0.194 mm)	512×512, (0.398 mm)
Active imaging area	$400\;\text{mm}(h)\times 300\;{\text{mm}}^{2}$	$397\;\text{mm}(h)\times 298\;{\text{mm}}^{2}$	$204\times 204\;{\text{mm}}^{2}$
Energy range	4−25 MV	40−150 kVp	40−225 kVp
Frame rate (Frames/sec)	1−10 fps	7.5 fps(1×1bin mode) 30 fps(2×2bin mode)	15 fps

The HD120 MLC of the Novalis Tx is designed for both stereotactic radiosurgery, as well as conventional radiation therapy. It consists of 120 leaves, which includes 64 2.5 mm inner leaves and 56 5 mm outer leaves. The dosimetric benefits of small leaf width have been previously demonstrated,^(^ ^26^ ^–^ ^33^ ^)^ and the central leaf width of 2.5 mm is close to the theoretically‐found optimum width (1.8 to approximately 2.0 mm for a 6 MV X‐ray beam).^(^ ^34^ ^)^ The HD120 MLC dosimetric properties were measured and found to have sharper penumbra than the micro MLC of the classic Novalis system.^(^ ^32^ ^)^

The design of good commissioning experiments and the development of comprehensive quality assurance (QA) tests are challenging for such a comprehensive system, especially in the context of SRS and SBRT treatments. General guidelines have been published in literature with regard to intensity‐modulated radiation therapy (IMRT)^(^ ^35^ ^,^ ^36^ ^)^ treatment planning systems,^(^ ^37^ ^)^ and multileaf collimators.^(^ ^38^ ^,^ ^39^ ^)^ Comprehensive linear accelerator QA and recommended tolerances have been published for both conventional and SRS/SBRT treatments.^(^ ^40^ ^,^ ^41^ ^)^

In this article, we provide technical guidelines for the Novalis Tx (NTX, Serial No. 3916) linear accelerator, from the time of acceptance testing to the first clinical treatment. The guidelines focus on methods for: (1) efficient and accurate beam data collection for commissioning treatment planning systems, including small field output measurements; (2) commissioning tests and routine daily and monthly QA of the HD120 MLC; (3) data collection for the baseline characteristics of the OBI and ETX in conjunction with the 6D robotic couch; and (4) end‐to‐end testing of the entire clinical process. The guidelines for commissioning and testing prior to clinical use follow the recommendations of the national practice guidelines referenced in this article including, among others, the American Association of Physicists in Medicine (AAPM) Task Groups: No. 142 (quality assurance for linear accelerators, Klein et al.^(^ ^41^ ^)^), No. 101 (stereotactic body radiotherapy, Benedict et al.^(^ ^42^ ^)^), AAPM Report No. 54 (stereotactic radiosurgery, Schell et al.^(^ ^43^ ^)^), and the ASTRO and ACR guidelines on stereotactic body radiotherapy (Potters et al.^(^ ^44^ ^,^ ^45^ ^)^).

Established from our clinical experience and collective patient data analysis, recommendations are provided for efficient use of the OBI and ETX localization systems for IGRT of rigid sites (e.g., brain and spine) and nonrigid regions such as the lung.

The technical, IT, and administrative resources need to support proper commissioning and ongoing routine QA efforts of the Novalis Tx technology at our institution are also discussed.

## II. MATERIALS AND METHODS

### A.1 Beam commissioning

Therapy outcome is directly related to the accuracy of radiation dose delivered to the patient. Thus, accurate baseline beam data measurements and overall commissioning of the linear accelerator is a crucial task, and is not without challenges. Small field dosimetry, required for commissioning of SRS and SBRT procedures, is especially difficult due to charged particle non‐equilibrium conditions, nonnegligible detector perturbation effects, and the enhanced influence of the finite source size.^(^ ^46^ ^)^ Studies have demonstrated significant differences between different radiation detectors (up to 10% to approximately 15%)^(^ ^46^ ^–^ ^54^ ^)^ and between different institutions, as well.^(^ ^53^ ^)^ Das et al.^(^ ^48^ ^,^ ^53^ ^)^ discussed various detectors and their pros and cons in the context of small field dosimetry. Alfonso et al.^(^ ^55^ ^)^ proposed a new formalism for small field reference dosimetry. The central leaf width of HD120 MLC is 2.5 mm. The required smallest field size to measure is $5\;\times \;5\;{\text{mm}}^{2}$ for iPlan 4.1 (BrainLAB, Feldkirchen, Germany) algorithms for SRS, and the smallest cone diameter is 4 mm. Following the suggestions of previous studies,^(^ ^56^ ^,^ ^57^ ^)^ the detector sensitive width should be no larger than 2 mm (the half the beam diameter in the beam's eye view) for such small field sizes. On the other hand, small diameter detectors have been shown to sometimes be problematic for large fields.^(^ ^49^ ^)^ Large variations were observed with different detectors with large field sizes. Because of such measurement difficulties, Monte Carlo (MC) simulations have been increasingly used as a promising alternative,^(^ ^46^ ^,^ ^58^ ^–^ ^60^ ^)^ but the MC algorithms must first be commissioned against measurements. It has been also demonstrated that the dose varies significantly with different jaw settings for a given MLC field size due to changes in the head scatter and related collimator exchange effects, which are enhanced at small field sizes.^(^ ^46^ ^)^ Thus, the measurements should strictly follow the jaw and MLC settings for the vendor‐specific dose calculation algorithm.

Although the required measured data is specific to the planning system to be commissioned, commonly required data includes nominal machine output, the leakage/transmission of beam shaping devices, depth profiles, lateral profiles, and relative scatter factors. Manufacturers typically provide guidelines and tolerances for their acceptance tests. However, in addition, the radiation beams should be properly commissioned by qualified medical physicists. The AAPM Task Group Report No. 45 provides general guidelines for linear accelerator acceptance testing and commissioning.^(^ ^61^ ^)^ AAPM Task Group Report No. 106 provides detailed recommendations on the choice of detectors, phantoms, and measurement procedures.^(^ ^49^ ^)^ In this section, we present measured beam data of our NTX, using four different detectors: stereotactic field diode (SFD), photon field diode (PFD), small cylindrical ion chamber (CC01), and a “medium‐sized” ion chamber (CC13) (Scanditronix Wellhofer, IBA Dosimetry America, Bartlett, TN, USA). The detector specifications are listed in Table 2.

**Table 2:** Ion chambers and diode detectors used in this study.

*Type*	*Model*	*Sensitive Volume*	*Diameter*	*Length/height*	*Misc.*
Cylindrical Ion Chambers	Scanditronix CC01	$0.01\;{\text{cm}}^{3}$	2 mm	3.6 mm	Steel central electrode
	Scanditronix CC13	$0.13\;{\text{cm}}^{3}$	6 mm	5.8 mm	C552 central electrode
	PTW PinPoint (31014)	$0.015\;{\text{cm}}^{3}$	2 mm	5.0 mm	Aluminum central electrode
	Standard Imaging Extradin A12	$0.65\;{\text{cm}}^{3}$	7.1 mm (outer shell)	12.9 mm (tip to center)	C552 central electrode
Diode Field Detectors	Scanditronix SFD	$0.03\;{\text{cm}}^{3}$	0.6 mm	0.06 mm	Unshielded
	Scanditronix PFD	$0.12\;{\text{cm}}^{3}$	2 mm	0.06 mm	Shielded

### A.2 MLC commissioning

The HD120 MLC consists of total 60 leaf pairs; 32 pairs of 2.5 mm central leaves and 28 pairs of 5 mm outer leaves, which produce a maximum beam width of 220 mm orthogonal to the leaf motion direction at the isocenter (Table 3). Along the leaf motion direction, both the maximum retracted and over‐travel distances are 200 mm. Therefore, the maximum field size the MLC can realize is $400\;\times 220\;{\text{mm}}^{2}$ at the isocenter. The maximum field size is limited to $320\;\times 220\;{\text{mm}}^{2}$ for intensity‐modulated treatment beams with dose rates up to 600 MU/min, and $150\;\times 150\;{\text{mm}}^{2}$ for 6 MV SRS treatment beams with dose rates up to 1000 MU/min.

**Table 3:** HD120 MLC specifications.

*Name*	*Value*
Number of leaves	60 pairs:
	−central: 2.5 mm×32 pairs(8 mm)
	−outer 5.0 mm×28 pairs(14 mm)
Max Retracted Position	200 mm
Max Extended Position	−200 mm
Max dynamic field size	$400\times 220\;{\text{mm}}^{2}$
Source to leaf bottom	538.3 mm
Interface mount to isocenter distance	415 mm
Max carriage speed	12 mm/sec
Max leaf speed	25 mm/sec

The commissioning of MLC commonly includes mechanical stability checks, leaf position verification, beam data acquisition, leaf transmission, leaf leakage, verification of beam penumbra, and the dynamic leaf gap test. AAPM Task Group No.142 provides guidance on the monthly and annual QA of the MLC systems for SRS treatments.^(^ ^41^ ^)^

The Winston‐Lutz (WL) test is used to check the MLC mechanical stability.^(^ ^62^ ^,^ ^63^ ^)^ A radio‐opaque ball (BB, 5 mm diameter) is placed at the isocenter and aligned to the linac light field cross‐hair. A small field opening ($10\;\times \;10\;{\text{mm}}^{2}$) is formed by the MLC and a film or EPID is placed at the beam exit with different combinations of gantry, collimator, and/or table angles (e.g., every 45°). The offset of BB center is measured with respect to the beam center for evaluation. The MLC mechanical stability check can be also accomplished using the spoke shot tests,^(^ ^64^ ^)^ also known as the “star‐shot” test. The test exposes films with various angles of collimator, gantry, and table rotations with a narrow slit radiation beam. The error should be no larger than 1 mm, according to the AAPM TG Report 142.^(^ ^41^ ^)^

The leaf positioning accuracy is usually checked by two tests — light and radiation field congruence test and a Picket Fence test. The former test compares the radiation field edge locations against those of the light field. A film is placed on the treatment table at the isocenter, the four light field edges are marked on the film surface with pressure or small pinprick holes, and the film is exposed to radiation. On the developed film, the radiation edges, or pinprick marks, should be in alignment with the light field edge within 1 mm on each edge (the tolerance based on the HD120 MLC specification). This test can also be performed using EPID with an object opaque for both light and radiation fields.^(^ ^65^ ^–^ ^67^ ^)^ The Picket Fence test can be also accomplished using a film or an EPID.^(^ ^68^ ^)^ While the radiation is on, a dynamic MLC plan is executed, which continuously moves the abutted leaf ends from one side to the other while halting at designated locations for several seconds. The “halted” locations, marked on the developed film or the EPID image, should appear in a straight line with the halt marks equidistant.

The leaf transmission, the leakage between two neighboring leaves (interleaf leakage), and the leakage between two abutting leaf ends can be measured with films.^(^ ^38^ ^)^ Two films are irradiated in a solid phantom ($100\;\times \;100\;{\text{mm}}^{2}$ jaw field, 50 mm depth); one with the MLC closed and the other with the MLC open. The films are converted to dose using a pre‐established optical density‐to‐dose response curve. The transmission and leakage are then measured as the ratios of the two doses. Alternatively, the mean transmission factor can be measured using either a Farmer‐type or parallel plate ion chamber large enough to span several leaves. The abutting leaf gaps are usually offset from the center so that they are not included in the measurement. The measured mean transmission should be less than 2%,^(^ ^39^ ^)^ and the variation from the baseline measurement should be less than 0.5%.^(^ ^41^ ^)^ The test should be carried out at different gantry/ collimator angles to check the effect of gravity.

Transversal profiles defined by MLC should be scanned to evaluate the beam penumbra. The beam penumbra from 80% to 20% becomes larger with greater depth and field size due to the increased percentage of scattered photons in the phantom which degrade the penumbra. Profile penumbra defined by the MLC should be incorporated into the beam modeling process, especially for SRS and small‐field SBRT treatment planning.

Each leaf of the HD120 MLC has a curved end. Thus, the physical leaf gap is different from the radiation leaf gap. Most treatment planning systems require this information for accurate dose calculation. The dynamic leaf shift is defined as the effective leaf shift from the nominal MLC leaf end position. The shift value can be measured by the sweeping gap technique.^(^ ^69^ ^)^

### B. Imaging tests

#### B.1 Image quality

**Figure 1:**
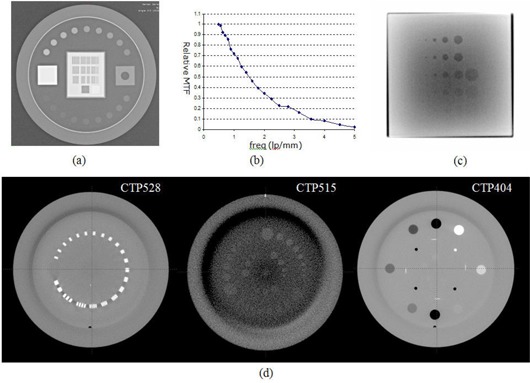
Phantom images acquired on NTX for image quality evaluation: (a) a Leeds phantom image for kV OBI spatial resolution measurement; (b) a relative MTF plot for image (a); (c) aluminum Las‐Vegas phantom image for MV contrast resolution measurement; and (d) CBCT axial slices of three Catphan 600 modules — CTP528 21 line/pair module, CTP515 low‐contrast module, and CTP 404 slice thickness, sensitometry, and pixel size module.

The primary goal of an IGRT system is to achieve accurate geometric localization. Obtaining images of high quality is central to this process. The image quality and quality assurance programs for the OBI (planar and CBCT) have been detailed in the studies by Yoo et al.^(^ ^70^ ^)^ and Bissonnette et al.^(^ ^71^ ^,^ ^72^ ^)^ Hyer et al.^(^ ^73^ ^)^ compared the qualities of two volumetric CBCT imaging systems, as well as the organ doses. Lee et al.^(^ ^74^ ^)^ assessed the image quality of the ETX system. Table 1 lists the technical specifications of the imaging detectors mounted on the NTX. Image quality is generally assessed by evaluating spatial and contrast resolution. Spatial resolution is commonly measured using a line‐pair phantom. Figure 1(a) shows an image of a line‐pair phantom (TOR 18FG, Leeds Test Objects Ltd., North Yorkshire, United Kingdom), acquired with the phantom placed on top of the NTX OBI kV detector surface (linac gantry angle: 90°, field size: $120\;\times 120\;{\text{mm}}^{2}$, source to detector distance: 1500 mm, and technique: 50 kVp, 80 mA, and 10 ms). The phantom contains 21 groups of line pairs with spatial frequencies ranging from 0.5 to 5.0 lp/mm. The spatial resolution can be quantified by visually determining the resolvable line pairs, or by plotting a relative modulation transfer function (RMTF) for more quantitative analysis,^(^ ^74^ ^–^ ^76^ ^)^ as shown in Figure 1(b). The contrast resolution is measured with a phantom with various contrast media. Figure 1(c) shows an MV portal image of an aluminum phantom, known as the “Las Vegas” phantom (Varian Medical Systems, Palo Alto, CA), which contains circular discs of different sizes and thicknesses. The visible circles are marked qualitatively. These methods of checking the image spatial and contrast resolution can be also applied to volumetric images.^(^ ^70^ ^,^ ^72^ ^)^ Figure 1(d) shows three CBCT slices of the Catphan 600 phantom (The Phantom Laboratory, NY). The first two images (CTP528 and CTP515 modules) are used for spatial and contrast resolution measurements. The third image (CTP404 module) is used for measuring the geometric accuracy (slice thickness and pixel size), as well as the HU accuracy. Another commonly measured quantity is the HU uniformity, which can be used to evaluate the CT cupping and capping artifacts^(^ ^77^ ^)^ using the CTP486 module (not included in Figure 1). The cupping artifact is associated with loss of pixel intensity at the central region of the phantom due to the beam hardening effect. It is typically corrected using measured data from cylindrical phantoms of different sizes.^(^ ^77^ ^)^ However, due to the fact that patients differ from the phantoms in size, there can be residual cupping artifacts. The capping artifacts arise from the over‐correction of the cupping artifacts.

#### B.2 Imaging system isocentricity

The AAPM task group report No. 142 recommends that the coincidence of isocenters of the imaging systems and the linac be checked daily. The tolerance of this test is 1 mm for SRS/SBRT treatments. Therefore, the isocenter offsets of the OBI planar kV/MV, volumetric CBCT, and ETX imaging systems should be measured accordingly. The offsets can be measured using any phantom with an internal BB with the crosshairs intersecting at the center of the BB. As shown in Section D below, it is not uncommon to see differences between the different imaging systems. Minimizing the difference between the imaging isocenters is of utmost importance, especially for SRS treatments for very small targets — for instance, those with diameters of only a few millimeters (e.g., trigeminal neuralgia), or for those that reside immediately adjacent to critical organs at risk.

**Figure 2:**
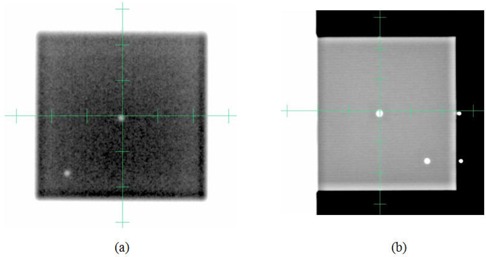
Example pair of (a) AP M V, and (b) right lateral kV images of OBI cube phantom after aligning the phantom with the room lasers. The isocenter BB offsets are measured using the digital graticules (green crosshairs).

The procedure, presented by Yoo et al.^(^ ^70^ ^)^ describes the OBI isocentric coincidence for planar kV and MV imaging systems. A BB phantom is setup to the linac isocenter and the BB offsets are measured on acquired planar X‐ray images. Measuring the BB offsets from four gantry angles (0°, 90°, 180°, and 270°) provides more information to assess accuracy than using two orthogonal angles. Figure 2 shows an example planar image pair, AP MV, and right lateral kV images of an OBI cube phantom with the phantom aligned to the room lasers. The BB centers are measured from the digital graticules, rendered as green crosshairs in the figure. The digital graticule offset from the beam central axis also needs to be evaluated and compensated for, as necessary.^(^ ^13^ ^)^

**Figure 3:**
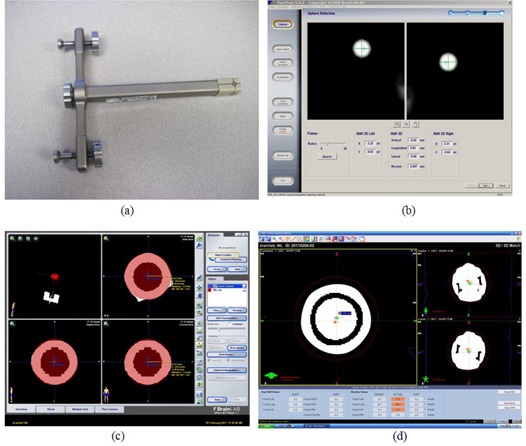
Measuring ETX and CBCT isocenter coincidence with the linac isocenter. First the BrainLAB Winston‐Lutz phantom (a) is aligned to the linac isocenter. The ETX Winston‐Lutz verification module detects the BB automatically on two planar images, and calculates the BB offsets from the imaging isocenter, as shown in (b). The CBCT imaging isocenter offset measurement includes (1) acquiring the BB CT image, (2) creating a treatment plan with an isocenter placed at the center of the BB (shown in (c)), (3) acquiring an CBCT image with the phantom setup to the linac isocenter, and (4) registering two BBs (as shown in (d)).

The ETX imaging isocenter can be measured using the BrainLAB Winston‐Lutz phantom (Figure 3(a)). With the phantom placed at the linac isocenter, two ETX planar images are acquired. The ETX Winston‐Lutz verification module automatically detects the BB on the images and calculates the BB location in 3D (Figure 3(b)), which is the offset of the linac isocenter from the ETX isocenter. This offset depends on the ETX isocenter calibration and should be less than 1 mm.

The CBCT imaging isocenter offset can also be measured using the BrainLAB Winston‐Lutz phantom. An example dataset is presented in Figure 3. First, the phantom was CT‐scanned with fine resolution (dimension: $512\;\times 512\;\times 134\;{\text{pixel}}^{3}$, voxel size: $0.2\;\times 0.2\;\times 0.4\;{\text{mm}}^{3}$) and the CT image was imported to the treatment planning system. A plan was then created with the isocenter placed at the center of the BB (Figure 3(c)). In order to align the plan isocenter accurately to the BB center, a spherical PTV was placed on the BB and a ruler tool was used to confirm the center alignment. Figure 3(d) shows a CBCT image acquired (FOV: $50\;\times 50\;\times 50\;{\text{mm}}^{3}$, voxel size: $0.1\;\times 0.1\;\times 1.0\;{\text{mm}}^{3}$) after the phantom was setup to the linac isocenter. The CBCT was manually registered to the planning CT by aligning two BBs (Figure 3(d)). The isocenter misalignment was then measured.

#### B.3 Localization accuracy of CBCT and ETX

In addition to verifying the coincidence of the imaging system and radiation isocenter, the entire localization process involves other steps, including image registration and couch correction, which may contribute to the overall uncertainty in the daily patient setup. It is important to estimate the localization accuracy after acceptance testing to understand the characteristics of each system.

For the isocentric localization error measurement, hidden target tests were performed using an anthropomorphic pelvis phantom with the plan isocenter set at the center of an internal BB. The phantom was positioned on the table with various initial setup errors, including rotational errors up to 2.5°. Each system was used to correct the initial errors through automatic image registrations.^(^ ^13^ ^)^ A pair of AP and LAT portal images were acquired using a $30\;\times \;30\;{\text{mm}}^{2}$ field size. The BB offset was measured with respect to radiation field center as the localization residual error. In addition, the differences in the rotational shifts calculated by the two registration algorithms were compared, and the accuracy of robotic couch angle corrections were measured. The latter quantities were measured by comparing the ETX couch correction angles with the actual table correction angles. The actual correction angles were calculated using eight embedded BBs, imaged on a pair of CBCT images acquired before and after table corrections.

### C. End‐to‐end testing

Radiation therapy, from simulation to treatment, involves several different processes, including CT simulation, treatment planning, target localization, and radiation delivery. Each of these processes has its own set of procedures. For instance, CT simulation has uncertainties associated with spatial resolution; treatment planning involves image registration, contouring of targets and normal tissues, and optimization/dose calculation. The combined uncertainties of these procedures and processes determine the overall end‐to‐end uncertainty. The aim of end‐to‐end testing is to estimate the overall combined accuracy for a given treatment scheme. The test is typically accomplished by applying the entire procedure to a phantom as if treating a patient.^(^ ^13^ ^,^ ^78^ ^–^ ^80^ ^)^ The delivered dose is measured using one or more dosimeters, including ion chambers, thermoluminescent dosimeters (TLDs), and films. Together with the dosimetry check, the geometric localization accuracy is commonly estimated using a hidden target test.^(^ ^13^ ^,^ ^78^ ^,^ ^81^ ^)^ The test is accomplished by imaging the isocenter BB, embedded in the phantom, using a small cone or square beam aperture after target localization, as has been described in the previous section.

**Figure 4:**
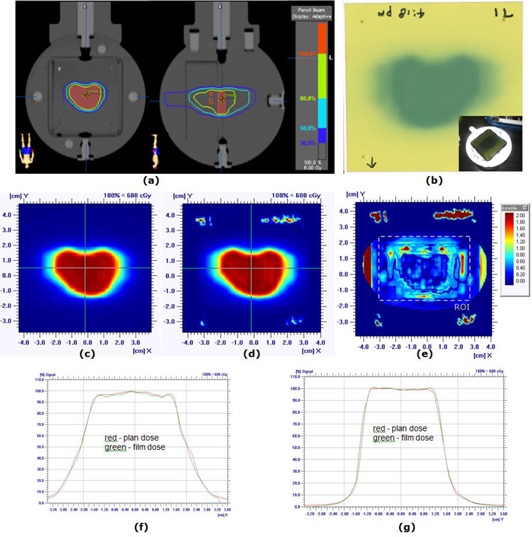
End‐to‐end test using Lucy 3D QA Phantom: (a) treatment plan developed on phantom CT scan, (b) an exposed irradiated GAFCHROMICTM EBT film, (c) plan dose distribution, (d) film dose distribution, (e) gamma index image with 3% dose difference and 1 mm distance to agreement (95% of pixels passed within ROI), (f) horizontal dose profiles, and (g) vertical profiles.

Figure 4 shows an end‐to‐end test using a SRS QA phantom (Lucy 3D QA, Standard Imaging Inc., Middleton, WI, USA) performed on the NTX. The phantom was CT‐scanned in 2 mm slice thickness^(^ ^82^ ^)^ with MRI Isocentric Volume Insert (REF 70107). The central, “mineral oil” filled object was contoured as the treatment target volume. The contour was transferred to a 2 mm slice thickness CT scan with Target/Treatment Verification Film Cassette (REF 70078). A treatment plan was developed with five IMRT beams around the contoured target (Figure 4(a)). The phantom was setup in the treatment room following the frameless SRS brain setup procedure in our clinic. The planned treatment fields were delivered and measured using a PinPoint 31014 ion chamber (PTW, Freiburg GmbH, Germany) as well as GAFCHROMICTM EBT2 (International Specialty Products, Wayne, NJ) films. Figure 4(b) shows an example irradiated film. Four films were irradiated and scanned to images using an Epson Expression 10000XL document flatbed scanner (Seiko Epson Corp, Nagano, Japan). The red and green channels were extracted and converted to dose using pre‐established calibration curves. All converted dose images were averaged.

**Figure 5:**
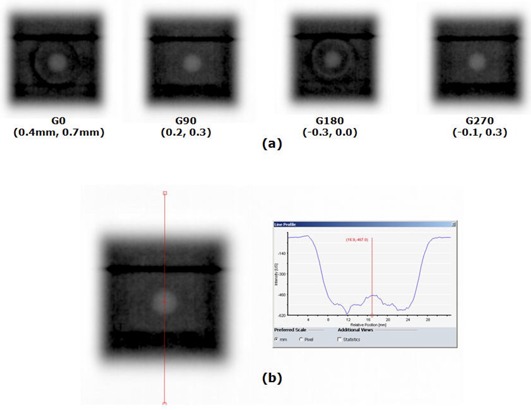
Hidden target test results for end‐to‐end test using Lucy 3D QA Phantom: (a) the brass ball phantom (REF50209) of 4 mm diameter was imaged on EPID with $20\;\times \;20\;{\text{mm}}^{2}$ MV radiation fields of gantry 0°, 90°, 180°, and 270°, with the numbers in the parentheses being the measured offsets in the horizontal and vertical directions, respectively; (b) the locations of field edges (50% intensity) and the brass ball center were determined using a line profile tool to measure the BB offset from the field center.

A hidden target test was performed with a 4 mm brass ball module (REF50209) inserted into the Lucy 3D QA Phantom. The ball phantom was imaged on EPID with $20\;\times \;20\;{\text{mm}}^{2}$, 6 MV radiation fields at gantry angles of G0°, G90°, G180°, and G270° (Figure 5). The field size was chosen such that the field edge determinations were not influenced by the BB signal. The offsets were calculated by locating the 50% intensity field edges and the center of the brass ball using a line profile tool, as shown in Figure 5(b).

### D. Clinical use of the Novalis Tx image guidance systems

We have treated over 400 SRS/SBRT cases since the installation of NTX at our institution (June 2008), and have developed detailed protocols for setup and localization of patients with rigid (brain and spine) and nonrigid (e.g., lung, liver) tumors. Procedures have been established based on results from phantom experiments, as well as our initial clinical experience with localization using ETX and OBI for patients with brain, spine, and lung tumors. In addition, in order to better understand the dosimetric impact of rotations in the localization process, we have retrospectively collected and analyzed ETX 3D/2D image registration results for 711 spine cases. For these cases, we have estimated dosimetric changes on target coverage and the spinal cord.^(^ ^83^ ^)^

### E. Support technology

Commissioning of a highly comprehensive linear accelerator, such as the NTX, requires careful coordination and planning of the entire radiation oncology team, including physicists, departmental administrators, and information systems/information technology (IS/IT) support. The responsibilities of various members of the team, appropriate staffing and administrative support for acceptance testing, commissioning, routine clinical use, and on‐going quality assurance of a linear accelerator being used to treatment with IMRT, IGRT, SRS and SBRT capabilities should follow the national guidelines and task group reports mentioned previously (AAPM Report No. 54 43, AAPM TG 101 42, AAPM TG‐142 41, and ASTRO/ACR guidelines reports 44, 45). From our experience, we have estimated the resources required for acceptance testing, commissioning, and on‐going quality assurance (see Results, Section F).

## III. RESULTS

### A. Beam commissioning

Machine output is generally measured in a water phantom at a depth of 5 or 10 cm with a $100\;\times \;100\;{\text{mm}}^{2}$ field size. Therefore, a calibrated standard‐sized $\left(0.6{\text{cm}}^{3}\right)$ cylindrical ion chamber is suitable for this measurement, and one should follow the detailed procedure described in AAPM Task Group Report No. 51.^(^ ^84^ ^)^ The measurement of the nominal linac output warrants special care because all other measurements are relative to this value. The nominal output of our NTX was 0.859 Gy/100 MU at 5 cm depth with 100 cm SSD, measured using Exradin A12 ion chamber (Standard Imaging, Middleton, WI).

**Figure 6:**
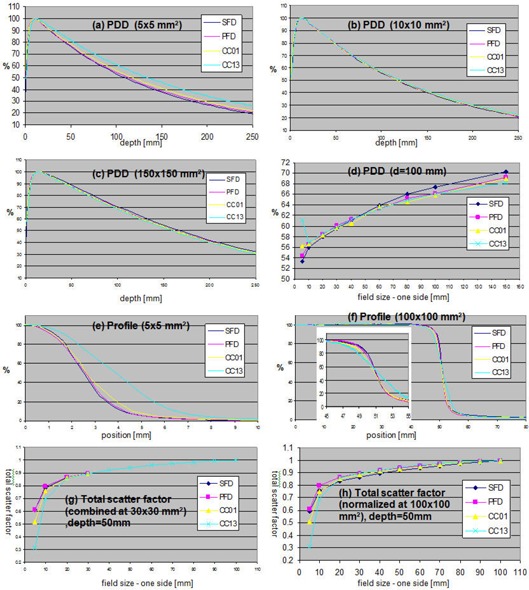
Measured NTX beam data using SFD, PFD, CC01, and CC13 detectors (Table 2): (a)–(c) PDD of fields $5\;\times \;5\;{\text{mm}}^{2}$, $10\;\times \;10\;{\text{mm}}^{2}$, and $150\;\times \;150\;{\text{mm}}^{2}$; (d) PDD at depth 100 mm of fields ranging $5\;\times \;5\;\sim \;150\;\times \;150\;{\text{mm}}^{2}$,; (e)–(f) cross‐plane profiles of 5 × 5 and $100\;\times \;100\;{\text{mm}}^{2}$ fields; (g) total scanner factors normalized at $30\;\times \;30\;{\text{mm}}^{2}$ and combined to CC13 results of larger fields;, and (h) total scatter factors normalized at $100\;\times \;100\;{\text{mm}}^{2}$. For PDDs and profiles of (a)–(f), the maximums were set to 100%.

The depth profiles, percent depth dose (PDD) or tissue maximum ratio (TMR), are measured in water along the central beam axis using various field sizes. Figure 6(a)–(c) show a set of PDD profiles of 6 MV SRS beam measured using four different detectors for field sizes of 5 × 5, 10 × 10, and $150\;\times \;150\;{\text{mm}}^{2}$. Figure 6(d) shows the PDD values at 100 mm depth for various field sizes. For all measurements, the ion chambers were mounted in the longitudinal direction, with the long axis of the chamber perpendicular to the beam central axis. The diodes were mounted in the vertical direction. The square field radiation beams were formed by jaws with the MLC retracted. The data shows that all PDD profiles are in good agreement (within 1% on average) for field sizes from $10\;\times \;10\;{\text{mm}}^{2}$ to $60\;\times \;60\;{\text{mm}}^{2}$. For field sizes larger than $60\;\times \;60\;{\text{mm}}^{2}$, the SFD overestimates the dose by approximately 2% over that of the CC13 ion chamber, which we consider the standard measurement device for larger field sizes. For the smallest field size, $5\;\times \;5\;{\text{mm}}^{2}$, the variation between detectors was large. The CC13 ion chamber has a large active diameter/volume relative to this small field size and, therefore, suffers from significant volume averaging. The CC01 ion chamber with an active length of 3.6 mm is also affected by volume averaging. The difference between two diode detectors was approximately 1%. The active diameter of the SFD is 0.6 mm and that of the PFD is 2 mm; therefore, the SFD is expected to be less influenced by the volume averaging effect. In addition, the effect of low energy scatter photons on the over‐response of the diode will be minimized at small field sizes. It should be noted the vertical detector travel alignment to the beam central axis is extremely important for small field size depth dose scans.^(^ ^46^ ^,^ ^49^ ^)^ The alignment should be confirmed by scanning in‐/cross‐plane profiles at different depths with a small field size. Some recent measurement device software tools, such as OmniPro‐Accept (IBA Dosimetry, Schwarzenbruck, Germany), are also capable of calculating the incident beam angles. The source‐to‐water surface distance (SSD) and the ion chamber detector geometrical centers were aligned to the water surface, following recommendations from AAPM Task Group Report No. 106.^(^ ^49^ ^)^ For diode detectors, the flat detector surfaces were aligned to the water surface. The effective point of measurement correction was applied for the ion chambers.^(^ ^84^ ^)^

The lateral profiles (in‐plane, cross‐plane, and/or diagonal profiles) are measured with different field sizes and at various depths. Figures 6(e) and (f) show cross‐plane half profiles of $5\;\times \;5\;{\text{mm}}^{2}$ and $100\;\times \;100\;{\text{mm}}^{2}$ field sizes measured at 15 mm depth. The profiles measured with the CC13 ion chamber show a large blurring of the penumbra due to the relatively large diameter of the sensitive volume (6 mm). A smaller degradation in the penumbra was observed for CC01 profiles (2 mm active diameter). The sharpest profile penumbra was observed for the SFD because of its small active diameter, 0.6 mm. However, SFD also shows a slightly higher response in the profile tail, outside of the field for the $100\;\times \;100\;{\text{mm}}^{2}$ field size. This is due to the over‐response of SFD to low energy scattered radiation, which increases with field size. Measurements of small field profiles require small volume detectors for sharp dose falloff in the penumbral region. If a diode is used, consideration must be given to the change in diode response with field size as a result of the over‐response of the diode to low energy scattered photons at larger field sizes. Ding et al.^(^ ^46^ ^)^ demonstrated good agreement between MC and stereotactic diode (SFD). Das et al.^(^ ^53^ ^)^ showed near‐identical profiles measured with film and diamond detector, outperforming a small size ion‐chamber $(0.015{\text{mm}}^{2})$ and Markus parallel‐plate chamber. For profiles with the sharpest penumbra, the authors recommended that the diamond detector be oriented such that its smallest dimension is parallel with the beam axis. Films have the highest resolution, well suited for small field profile measurements.^(^ ^85^ ^)^ However, film requires a well‐established dosimetry protocol for minimizing measurement uncertainties.^(^ ^86^ ^)^ External beam therapy (EBT/EBT2, International Specialty Products, Wayne, NJ) films are self‐developing (thus, chemical film processors are not required), relatively independent of energy, and are nearly tissue‐equivalent. They have been used in several applications,^(^ ^87^ ^)^ as well as for small field dosimetry.^(^ ^85^ ^,^ ^88^ ^,^ ^89^ ^)^ They are often scanned to 48 bit RGB images using a desktop scanner, and the red channel is used for dosimetry because of its highest sensitivity per a given fractional radiation dose.^(^ ^90^ ^)^ However, the scanner light source and detector elements are nonuniform in response, which needs to be compensated for. It is recommended that the profile be measured along scan direction (orthogonal to the CCD array line), in which case the correction is smaller (~1%).^(^ ^91^ ^,^ ^92^ ^)^

The total scatter factor, also known as the output factor, is the ratio of the dose rate for a given field size to that of the reference field size (typically $100\;\times \;100\;{\text{mm}}^{2}$) measured at a reference depth.^(^ ^93^ ^)^ Figure 6(g) shows the total scatter factor curves measured at 50 mm depth for field sizes ranging from $5\;\times \;5\;{\text{mm}}^{2}$ to $100\;\times \;100\;{\text{mm}}^{2}$. For fields larger than $30\;\times \;30\;{\text{mm}}^{2}$, CC13 results were used. For smaller field sizes, the measurements for each detector were normalized by the measurements of the $30\;\times \;30\;{\text{mm}}^{2}$ field size, and multiplied by the total scatter factor of the CC13 ion chamber at $30\;\times \;30\;{\text{mm}}^{2}$, normalized to the $100\;\times \;100\;{\text{mm}}^{2}$ field size as follows:^(^ ^79^ ^)^

(1) $$\frac{D(f)}{D(30\times 30)}\times \frac{CC13(30\times 30)}{CC13(100\times 100)}$$

In the equation, *D(f)* represents a measured dose by a detector for a field size *f*. As shown in Figure 6(g), the CC13 and CC01 show lower output scatter factors for small field sizes due to volume averaging, and the two diode detectors show good agreement. Choosing $20\;\times \;20\;{\text{mm}}^{2}$ as the intermediate filed size was found to produce similar results (not shown in the figure). Figure 6(h) shows the output factors normalized to the $100\;\times \;100\;{\text{mm}}^{2}$ field size. This method is generally not recommended due to the over‐response of small detectors at large field sizes (over‐response to low‐energy photons and detector stem effect). The results are included here for reference. The results are in agreement with those of the Das et al. study.^(^ ^48^ ^)^

### B. MLC commissioning

**Figure 7:**
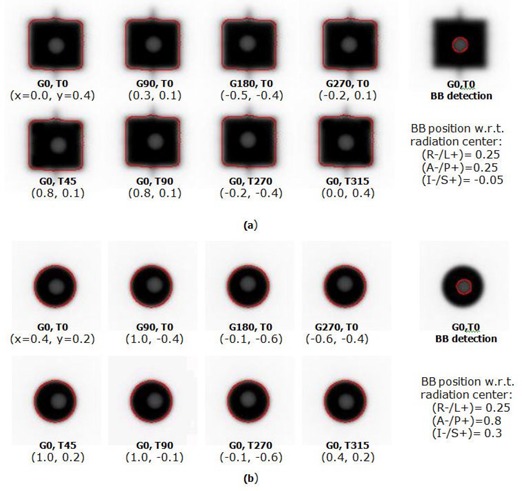
EPID‐based Winston‐Lutz tests using: (a) $15\;\times \;15\;{\text{mm}}^{2}$ MLC, and (b) 12.5 mm cone. The numbers followed by G and T are, respectively, the gantry and table angles in degrees.

An example set of MLC‐ and cone‐based Winston‐Lutz images is given in Figure 7. The MLC field size was set to $15\;\times \;15\;{\text{mm}}^{2}$ (jaw: $20\;\times \;20\;{\text{mm}}^{2}$) and the cone diameter to 12.5 mm. The field aperture was larger than that of typical film‐based WL tests ($10\;\times \;10\;{\text{mm}}^{2}$ for MLC and 7.5 mm for cone) so that the automatic detection algorithm was able to better distinguish the BB and the field edges. The red lines in the figure are the detected BB and field edges. The numbers followed by ‘G’ and ‘T’ refer to the gantry and table angles in degree, respectively. The numbers in parentheses (x, y) are the automatically detected BB offsets in the left(−)/right(+) and up(−)/down(+) directions, respectively, at the isocenter level. The calculated BB location with respect to the radiation isocenter is also presented in the lower right corner of each sub‐figure. They were calculated using four gantry rotation images with table zero as follows:

(2) (R−/L+)=(G0.x−G180.x)/2

(3) (A−/P+)=(G90.x−G270.x)/2

(4) (I−/S+)=(G0.y+G90.y+G180.y+G270.y)/4

This calculated BB location can be used to align the BB to the radiation isocenter more accurately. By performing these two tests without moving the BB, one can compare the radiation beam central axis misalignments between the MLC and cone. Two monitor units (MU) were used for each image acquisition.

**Figure 8:**
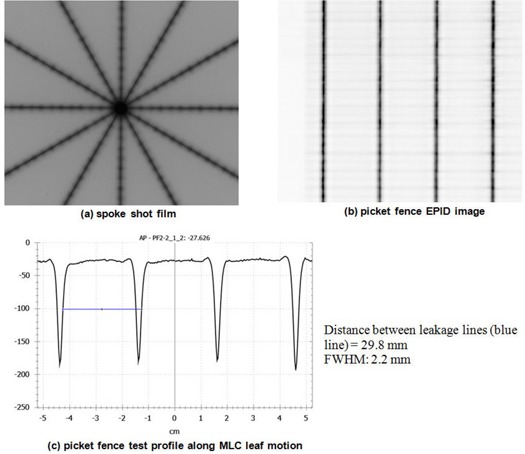
Examples of: (a) spoke shot film, (b) Picket Fence EPID image (25 MU, 400 MU/min, integration mode, and 3 cm interline distance at isocenter level), and (c) Picket Fence test profile along the MLC leaf motion and FWHM measurements. Blue line=distance measurement between leakage lines.

Figure 8(a) shows an example spoke shot of a film exposed at various collimator angles with the MLC closed. All lines were produced with MLC leaves closed. The lines produced by radiation should intersect within a circle of 1 mm radius.^(^ ^41^ ^)^ Figure 8(b) shows an EPID image of the Picket Fence test. To cover the entire area of the MLC motion, two images were acquired, one image for each MLC bank. The image of each bank contained five lines (at which the MLC was halted) with 30 mm interline distance. One image was acquired with 25 MU at 400 MU/min dose rate. Both of the test results are first evaluated visually, and further quantitative analysis is carried out if misalignments in the MLC positions are noted on the image. Figure 8(b) presents a profile of the Picket Fence test image of Figure 8(b). The profile was exported as an image and analyzed using ImageJ, a public domain image processing software developed at the National Institutes of Health (http://rsbweb.nih.gov/ij). For the distance measurement in mm, the pixel pitch was calibrated using the x‐axis on the profile image. The measured distance, shown as a blue line in the figure, was 29.8 mm and the FWHM was 2.2 mm. Chang et al.^(^ ^68^ ^)^ compared the film and EPID based picket fence tests and described quantities to be measured.

The measured mean MLC transmission, the ratio of the dose measured with MLC closed to that with a $100\;\times \;100\;{\text{mm}}^{2}$ MLC‐defined field, was 1.17% at zero gantry angle for the 6 MV SRS beam of our NTX unit.

**Figure 9:**
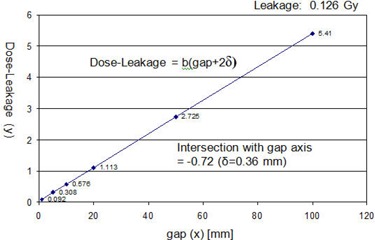
Dynamic leaf gap test for the HD120 MLC on the NTX; SSD = 980 mm, depth = 20 mm, and jaw $\text{field}=100\times 100\;{\text{mm}}^{2}$. The nominal gaps were 1, 5, 10, 20, 50, and 100 mm. The line intersection on the x‐axis was −0.72 mm, and the dynamic leaf shift (δ), the negative half of the intersection, was 0.36 mm.

Figure 9 shows a result of dynamic leaf gap test using the equation:

(5) $$D-D_{leak}=b(gap+2\delta )$$

Doses, *D*, were measured in water using an ion chamber with a set of MLC dynamic plans sweeping from one end of the field to the other while maintaining a constant gap (nominal gap). The nominal gaps were 1, 5, 10, 20, 50, and 100 mm. The measured doses with MLC leakage subtracted (${\text{D‐D}}_{\text{leak}}$) were plotted as a function of the nominal gap. Extrapolating and finding the line intersection on the gap (x) axis gave the zero dose gap of −0.72 mm. The negative half of this value is the dynamic leaf shift: δ = 0.36 mm. The leakage dose ($D_{\text{leak}}$) was 0.126 Gy, measured using an ion chamber with all the MLC leaves closed and offset by 50 mm from the center line.

### C. Imaging tests

#### C.1 Image quality

Figure 1(b) is the RMTF of image Figure 1(a), which was normalized to the MTF of 0.5 lp/mm. The measured frequency of 50% RMTF ($f_{50}$) was 1.5 lp/mm.

#### C.2 Imaging system isocentricity

The ETX measured isocenter offsets (mean ± std) were 0.0±0.2 mm, −0.7±0.2 mm, and −0.6±0.2 mm in the L(+)/R(−),A(−)/P(+), and S(+)/I(−) directions, respectively, from eight days of X‐ray images. The CBCT isocenter offsets (mean ± std) were 0.1±0.4 mm, 0.4±0.5 mm, and 0.4±0.4 mm in the L(+)/R(−),A(−)/P(+), and S(+)/I(−) directions, respectively, from CBCT images of 28 different days. The offsets of the ETX and CBCT imaging systems were measured with respect to the Winston‐Lutz BB, which can be correlated to the radiation isocenter position.

#### C.3 Localization accuracy of CBCT and ETX

**Figure 10:**
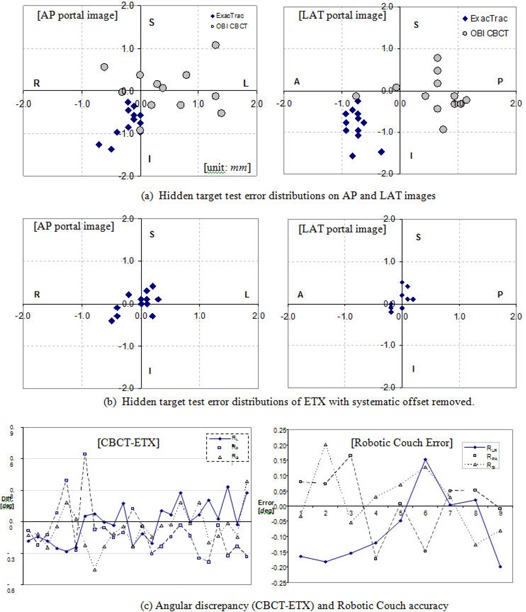
Localization accuracy of ETX and CBCT: (a) hidden target test error distribution on AP and LAT portal images (units in mm), (b) hidden target test error distribution of ETX with systematic offsets removed (μ ± σ = 0.0±0.3 mm for L/R, 0.0±0.1 mm for P/A, and 0.1±0.2 mm for S/I), and (c) angular discrepancy between 3D/2D ETX and 3D/3D CBCT image registration algorithms (left) and robotic couch error estimated from 8 embedded BBs on 9 pairs of pre/ post CBCT images (right).

Figure 10 shows a set of localization accuracies of the ETX and CBCT systems, as well as the robotic couch accuracy. Figure 10(a) represents the error distributions of 12 measurements on the anterior–posterior(AP) and lateral (LAT) image planes. The mean and standard deviations are listed in Table 4. Data acquired with the ETX system, marked as diamonds, show smaller random distributions with small (< 1 mm in one direction) but measurable systematic error, relative to the CBCT system. It was possible to correct the ETX systematic error by calibrating the system at the offset location, thereby compensating for the systematic error. The corresponding error distributions are shown in Figure 10(b).^(^ ^13^ ^)^ The CBCT system residual error shows a larger random distribution with smaller systematic error relative to the ETX system. We speculate the larger random error may be due to the limited table correction resolution.^(^ ^13^ ^)^ The largest difference between the two systems occurred in the anterior–posterior direction (1.3±0.6 mm), while the individual system error was within acceptable error range. Figure 10(c) left shows the differences in the rotational shifts calculated by the two registration algorithms. The differences were minimal (0.1±0.2°). The table angle correction was accurate with a maximum of only 0.2° error as shown in Figure 10(c) right. The error mean and standard deviations were −0.1±0.1° around the left/right axis ($R_{\text{LR}}$) and 0.0±0.1° about the posterior/anterior ($R_{\text{PA}}$) and superior/inferior axes ($R_{\text{SI}}$), respectively.

**Table 4:** Isocenter error statistics of ETX and OBI CBCT (unit: mm).

	*x L(+)/R(−)*	*y P(+)/A(−)*	*z S(+)/I(−)*	*Vector Length*
ETX	−0.2±0.2 mm	−0.8±0.2	−0.8±0.4	1.2±0.3
OBI CBCT	0.5±0.7	0.6±0.5	0.0±0.5	1.1±0.4
OBI CBCT‐ETX	0.7±0.7	1.3±0.6	0.8±0.5	1.9±0.5

### D. End‐to‐end testing

The isocenter doses measured by the ion chamber are summarized in Table 5. The measured dose differed from the plan by 2.1% for one beam, and the overall difference was 0.8%. The EBT2 film dosimetry results are shown in Figures 4(c)–(g). The plan and film dose distributions are qualitatively similar. The gamma index image in Figure 4(e), calculated with 3% dose difference and 1 mm distance to agreement, indicates that the differences exist in and around the target boundaries. For the given gamma criteria, 95% of pixels had passed (gamma < 1.0) within the defined region of interest (the dashed line in Figure 4(e)). The measured film dose at the isocenter was within 0.4% of the planned dose. Figures 4(f) and (g) show the horizontal and vertical dose profiles, respectively. The hidden target test results are shown in Figure 5. The measured horizontal and vertical offsets were 0.4 mm and 0.7 mm for G0°, 0.2 mm and 0.3 mm for G90°, −0.3 mm and 0.0 mm for G180°, and −0.1 mm and 0.3 mm for G270°.

**Table 5:** End‐to‐end test isocenter dose measurement result using a PTW PinPoint 31014 ion chamber.

*Beam*	*1*	*2*	*3*	*4*	*5*	*Total*
Plan Dose (cGy)	142	122	114	110	108	596
Chamber Reading (nC)	0.5604	0.4888	0.4557	0.4396	0.437	2.3815
Chamber Dose (cGy)	141.4	123.3	115.0	110.9	110.3	600.9
Chamber Dose ‐ iPlan Dose (%)	−0.4%	1.1%	0.9%	0.8%	2.1%	0.8%

### E. Clinical use of the Novalis Tx image guidance systems

**Figure 11:**
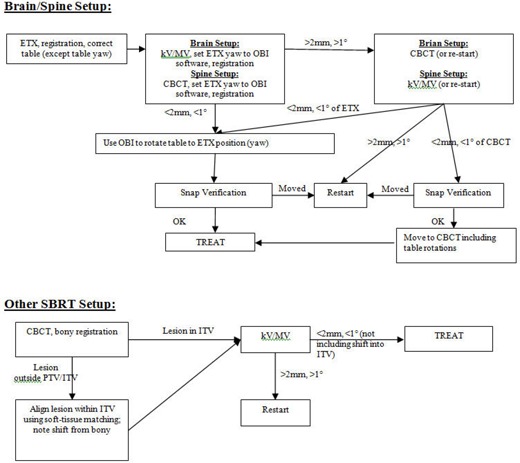
Patient setup procedures for brain, spine, and other SBRT cases using ETX, OBI, and CBCT imaging on the NTX.

Figure 11 shows the established patient setup procedures, which are currently practiced in our clinic. Modifications should be made for clinical flow at individual institutions.

For patients with “rigid” tumors in the brain and spine, the ETX system is used for the initial setup and localization. This system has been shown in numerous studies to work accurately for rigid targets, with ample bony anatomy to facilitate accurate image registration.^(^ ^5^ ^,^ ^13^ ^,^ ^94^ ^–^ ^97^ ^)^ Following the initial ETX‐based setup, additional images are acquired with the OBI. The detailed procedure, as shown in Figure 11, is as follows:

Step 1 (primary imaging). Set up patient using ETX, adjusting all translational shifts and two robotic couch rotations (roll and pitch). The table angle (yaw) (i.e., the angle around vertical axis) is not adjusted at this time.

Step 2 (verification imaging). On the OBI workstation, take a pair of AP MV and LAT kV images for brain, or take a CBCT image for spine. Perform automatic image registration. Then, manually set the table angle of ETX, which was not corrected in the previous step, in the OBI software. Verify registration and make manual adjustments if necessary. If the OBI shifts are < 2 mm and < 1° in all dimensions, reset all OBI shifts to zero except for the table angle. Correct the table angle using OBI. Using the OBI for the table angle correction ensures that the table angles of all treatment fields are updated automatically.

Step 3 (secondary verification imaging). If the first verification imaging disagrees (> 2 mm or > 1°) and at the physician's discretion, either a second set of verification imaging is performed (CBCT or kV/MV), or restart the setup process from the beginning. If the second verification image is taken and is within the tolerance of ETX, the patient is positioned according to ETX, or, if within the tolerance of CBCT or kV/MV images, the patient is set up to the CBCT location. If all three images do not agree to each other, restart the setup. It should be noted that although we have this process in place, neither imaging system has, to date, been outside of tolerance from ETX (i.e., a second verification has never been required).

Step 4. Before treatment commences, the ETX snap verification is performed. If it failed, it is assumed that patient has moved and the patient setup process starts over. Snap verification images are also taken at other points of time over treatment when necessary.

The second flow chart in Figure 11 is for nonrigid, extracranial lesions other than the spine cases. In these cases, the OBI‐based CBCT is the primary imaging modality because volumetric images are necessary to visualize the tumor; soft tissue contrast is highly limited on planar X‐rays. Visualization of the tumor is especially important under circumstances where tumor motion can be significant (e.g., in the thoracic region). The detailed localization procedure is as follows:

Step 1 (primary imaging). Set up patient based on tattoos or any setup procedure from the simulation/treatment planning stage. Take a CBCT image and align with the reference CT using automatic registration algorithm. Then, verify bony alignment and adjust manually, if necessary. If the lesion is visible and falls outside the ITV contour, adjust the fusion manually such that the contour encompasses the lesion. Once the fusion is approved by physician, correct the table shifts.

Step 2 (verification imaging). Take a pair of AP MV and LAT kV images for verification. Perform automatic fusion to the CT DRR images. Make any necessary manual adjustments. If the fusion result is within the tolerance of 2 mm and 1° (not including the additional shifts made for the soft tissue matching in the previous step), treatment can commence at the CBCT position. Otherwise, restart the setup. This step serves as a failsafe for any possible fault of the table correction in Step 1, as well as any systematic error in the CBCT system.

Table 6 shows the differences between the primary and verification imaging modalities following our routine clinical localization procedure described above (see Figure 11). The smallest difference was observed between the MV/kV pair and the CBCT image. This is probably because the two imaging modalities share the same isocenter. The difference between ETX and CBCT (the third column of Table 6) is the largest. The disagreement is highest along the anterior–posterior direction. This result agrees with that of the phantom study shown in Table 4, and indicates a systematic difference due to misalignment between the OBI and ETX system isocenters. We have described this issue in a recent publication (see Kim et al.^(^ ^13^ ^)^). We speculate that possible causes of such differences include: (1) small miscalibration of the CBCT system, and (2) centroid detection error of the infra‐red markers on the ETX isocenter calibration phantom. The vendor (BrainLab) is aware of this systematic offset, and a new version of software is soon to be released that allows users to calibrate ETX isocenter directly to the radiation isocenter using the Winston‐Lutz phantom. We have incorporated the measurements of the ETX and CBCT system isocenter offsets in our routine daily QA procedure in an effort to minimize the systematic difference between the two systems.

**Table 6:** Primary and verification imaging differences.

	ETX → MV/kV	ETX → CBCT	CBCT → MV/kV
	(N=63)	(N=81)	(N=47)
AP (y)	0.6±0.8 mm	1.1±0.7 mm	−0.2±0.7 mm
SI (z)	0.8±0.9 mm	0.8±0.8 mm	0.1±0.7 mm
Lat (x)	0.3±0.8 mm	0.2±0.7 mm	0.0±0.7 mm
Rot (Ry)	0.0±0.1 deg	−0.1±0.4 deg	0.0±0.0 deg
Sites	Brain/Spine	Brain/Spine	Lung/Pelvis

Note: N = number of localizations performed. Read “→” as “followed by”.

**Figure 12:**
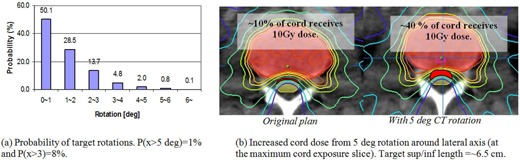
Dosimetric effect of rotational error: (a) rotational occurrences of 711 ETX 3D/2D image registrations (combined in all directions), and (b) the increased cord maximum dose from a simulated 5° rotation about the lateral axis for an elongated target (the PTV encompassed two vertebral bodies).

Figure 12(a) shows the probability of rotational occurrences estimated from 711 ETX 3D/2D image spine registrations. The results of three different axes were combined into one plot because there were no significant axis‐dependent differences. With the assumption that the image registration uncertainty is small, the probability of target rotations greater than 5° was approximately 1%, and that of larger than 3° was approximately 8%. Although, on average, the overall dosimetric effect of rotations is small, we have shown that care must be taken especially when treating elongated target volumes.^(^ ^83^ ^)^ Figure 12(b) illustrates an example where the planning target volume encompasses two vertebral bodies. In the original plan, the cord volume of 10 Gy was 10%, but it increased to 40% at the maximum impinging slice, which would not be clinically acceptable. Therefore, it is recommended that rotational errors be corrected.

### F. Support technology

Table 7 shows the tasks and the corresponding full time equivalent (FTE) staffing estimated for acceptance, commissioning, and routine QA of our NTX based on our experience.

**Table 7:** Tasks and estimated FTE (full‐time equivalent) for acceptance, commissioning, and routine QA of a Novalis Tx, based on our experience.

*Task*	*Estimated Time Commitment*	*Estimated FTE*
Customer acceptance procedure of the linac, OBI, and ETX image guidance systems	4–5 weeks	1.0 Physicist working with engineers
Measurements for beam modeling of the iPlan (Brainlab) TPS	2–3 weeks	2.0 Physicists; additional 0.5 physicist for checking, processing of the beam data for beam modeling
Measurements for beam modeling of the Eclipse (Varian) TPS	2–3 weeks	2.0 Physicists; additional 0.5 physicist for checking, processing of the beam data for beam modeling
Beam modeling for iPlan	1–2 weeks	Beam modeling for iPlan is performed by BrainLAB off‐site
Beam modeling for Eclipse	1 week	1.0 Physicist; additional 0.5 physicist for independent review of the beam modeling process
Verification measurements of the iPlan and Eclipse TPS beam models and additional testing of the linac, including the isocentricity, MLC transmission, leakage, etc.	2–3 weeks	2.0 Physicists
Beam calibration calculations and measurements; independent TLD verification of the linac calibration	1 week	2.0 Physicists
IT/IS support for supporting network, TPS, and R/V databases	1 week	1.0 IT/IS‐trained person
Routine monthly QA, including mechanical and output/calibration checks of the linac and QA of the image‐guidance systems	2 days (16 hrs) per month	1.0 Physicist
Routine annual QA including mechanical and output checks of the linac and QA of the image‐guidance systems	2 weeks (80 hrs) per year	1.0 Physicist + 0.25 Physicist for independent verification

## IV. DISCUSSION

### A. Limited maximum field size of HD120 MLC

**Figure 13:**
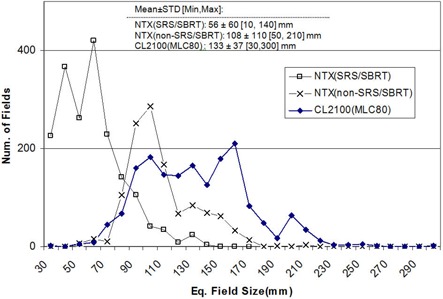
Statistics of treatment field sizes for two years (2009–2010) on NTX and CL2100. The number of patients were 214 for NTX(SRS/SBRT), 144 for NTX(non‐SRS/SBRT), and 425 for CL2100(MLC80). The values of mean, standard deviation, minimum, and maximum are given in the mid‐top area of the plot. Only the first treatment sessions were included for analysis.

Wu et al.^(^ ^32^ ^)^ demonstrated the dosimetric benefit of fine leaf width (2.5 mm) of HD120 MLC for small and complex‐shaped SRS targets. However, the maximum field size is limited to $400\;\times \;220\;{\text{mm}}^{2}$, which is smaller than that of conventional MLCs designed for non‐SRS/ SBRT treatments ($400\;\times \;400\;{\text{mm}}^{2}$). Therefore, it is feasible that non‐SRS/SBRT treatments with large field sizes may not be treatable on the NTX. For better understanding, we compared the treated field sizes of the NTX of this study with a Varian CL2100 (Varian Medical Systems, Palo Alto, CA) linear accelerator at the same department. The CL2100 unit is equipped with a conventional MLC (MLC80, Varian Medical Systems) that has 80 1 cm leaves with $400\;\times \;400\;{\text{mm}}^{2}$ maximum field size. Figure 13 shows the statistics of treatment field sizes for two years (2009–2010) on the NTX and CL2100. The number of patients were 214 for NTX(SRS/ SBRT), 144 for NTX(non‐SRS/SBRT), and 425 for CL2100(MLC80). The mean, standard deviation, minimum, and maximum equivalent field sizes are presented in the top area of the plot. As expected, the equivalent field sizes of SRS/SBRT cases, treated on NTX, were the smallest (mean μ = 56 mm). The equivalent field sizes of non‐SRS/SBRT cases treated on NTX were smaller than those of CL2100 (μ = 108 mm vs. 133 mm). Approximately 6% of total non‐SRS/ SBRT cases (34 out of 566 cases) exceeded the maximum field width (220 mm) of the HD120 MLC in the Y direction. Approximately 2% (12 cases) were found to be treatable on NTX after plan modifications including collimator/gantry angle adjustments. The remaining cases (22 cases, 4%), requiring large and irregular field apertures, were found to exceed the maximum field size of the HD120 leaf MLC.

### B. Cone‐based treatments

Cone‐based SRS treatments are rare at our institution. There were two cases on the NTX over two years (2009–2010), both of which were for frame‐based trigeminal neuralgia treatments using a 4 mm cone. The prescription doses were 70 Gy and 80 Gy for these cases, and both were delivered in a single fraction. Special attention must be given to the jaw size as well as the mounted cone diameter. One must refer to the recommended jaw sizes published by the vendor. For the cone recognition, the vendor recently released a barcode conical collimator verification system (BCCV, Varian Medical Systems, Palo Alto, CA), which allows recognition of mounting and dismounting of a specific size cone, and includes appropriate hardware interlocks. The BCCV is a mandatory upgrade for cone‐based SRS treatments.

### C. Jaw calibration verification

Ding et al.^(^ ^46^ ^)^ demonstrated strong influence of the jaw setting on the beam particle fluence, especially for small field radiation beams. Therefore, as described in the Section II. A.1, the measurements for treatment planning system commissioning must follow the vender‐specific jaw and MLC settings. In addition, the jaw calibration needs to be carefully verified.^(^ ^69^ ^)^ The verification can be accomplished using light/graph paper comparison tests, light/radiation field congruence tests, and full with half maximum (FWHM) measurements from profile scans. The measured quantities should be within 1 mm on each edge.

### D. Energy modes of the NTX

The NTX at our institution has two other photon energy modes in addition to the 6 MV SRS; 6 MV and 18 MV. We use 6 MV SRS mode for SRS/SBRT treatments, 6 MV and 18 MV for other regular treatments. The 6 MV SRS mode has a different flattening filter, designed for smaller field SRS treatment beams. In our institution, we use iPlan (BrainLAB, Feldkirchen, Germany) and Eclipse (Varian Medical Systems, Palo Alto CA) treatment planning systems for SRS/SBRT treatment planning. For the treatments involving the thoracic region, only iPlan Monte Carlo and Eclipse AAA (Anisotropic Analytical Algorithm) dose calculation algorithms are used for more accurate dose estimation. Each calculation algorithm from different vendor has different beam data requirements for commissioning. One must consult with the specific vendor‐provided documentation, as well as the national standard‐of‐care procedure guidelines and task group reports. In terms of the procedures and tolerances for the NTX commissioning, there is no special difference in procedures among available photon energies.

### E. Novalis classic vs. Novalis Tx

The NTX is a combination of the classic Novalis and Trilogy system with HD120 MLC. In terms of the clinical usage, the improvements of NTX in comparison to the classic Novalis system include: (1) the addition of the volumetric CBCT imaging system, (2) a larger maximum field size with the HD120 MLC relative to that of the m3 micro‐MLC (BrainLAB, Feldkirchen, Germany), (3) larger gantry rotation clearance, and (4) higher dose rate (600 MU/min). First, the visibility of soft tissue on CBCT images has expanded the treatable SBRT sites to soft tissue areas including lung and liver, which are difficult to localize on planar ETX X‐ray images. Second, the larger aperture of HD120 MLC allows more diverse treatments including non‐SRS/ SBRT treatments. Third, the gantry rotational clearance of NTX (41.5 cm) is larger than that of the classic Novalis (35.5 cm), because the m3 micro‐MLC is add‐on tertiary, while HD120 MLC is enclosed within the gantry. Consequently, larger patients with lesions located in the posterior aspect are difficult to set up on the classic Novalis table, and the freedom on gantry angle selection is limited. Finally, the high dose rate for 6 MV SRS beam (1000 MU/minute) allows quicker treatment delivery, which may help minimize intratreatment patient motion.

## V. CONCLUSIONS

We have described technical guidelines for the Novalis Tx linear accelerator from the time of acceptance testing to the first clinical treatment for SRS and SBRT, focusing on the methods for basic beam data measurement, commissioning of a HD120 MLC, quality assurance of the image‐guided systems, and end‐to‐end testing. The technology and technical staff necessary to support the program at our institution has also been discussed. In addition, we have presented the clinical IGRT procedures for different treatment sites established in our clinic based on phantom studies and our initial clinical experience. These methods and procedures should be adapted based on the clinical circumstances at individual institutions. We have demonstrated the Novalis Tx to be a robust system, with the image guidance and MLC requirements to treat a wide variety of sites, in addition to achieving the requirements for highly accurate delivery of SRS and SBRT‐based treatments.
